# Nature's Swiss Army Knives: Ovipositor Structure Mirrors Ecology in a Multitrophic Fig Wasp Community

**DOI:** 10.1371/journal.pone.0023642

**Published:** 2011-08-31

**Authors:** Mahua Ghara, Lakshminath Kundanati, Renee M. Borges

**Affiliations:** 1 Centre for Ecological Sciences, Indian Institute of Science, Bangalore, India; 2 Department of Mechanical Engineering, Indian Institute of Science, Bangalore, India; Lund University, Sweden

## Abstract

**Background:**

Resource partitioning is facilitated by adaptations along niche dimensions that range from morphology to behaviour. The exploitation of hidden resources may require specially adapted morphological or sensory tools for resource location and utilisation. Differences in tool diversity and complexity can determine not only how many species can utilize these hidden resources but also how they do so.

**Methodology and Principal Findings:**

The sclerotisation, gross morphology and ultrastructure of the ovipositors of a seven-member community of parasitic wasps comprising of gallers and parasitoids developing within the globular syconia (closed inflorescences) of *Ficus racemosa* (Moraceae) was investigated. These wasps also differ in their parasitism mode (external versus internal oviposition) and their timing of oviposition into the expanding syconium during its development. The number and diversity of sensilla, as well as ovipositor teeth, increased from internally ovipositing to externally ovipositing species and from gallers to parasitoids. The extent of sclerotisation of the ovipositor tip matched the force required to penetrate the syconium at the time of oviposition of each species. The internally ovipositing pollinator had only one type of sensillum and a single notch on the ovipositor tip. Externally ovipositing species had multiple sensilla types and teeth on their ovipositors. Chemosensilla were most concentrated at ovipositor tips while mechanoreceptors were more widely distributed, facilitating the precise location of hidden hosts in these wasps which lack larval host-seeking behaviour. Ovipositor traits of one parasitoid differed from those of its syntopic galler congeners and clustered with those of parasitoids within a different wasp subfamily. Thus ovipositor tools can show lability based on adaptive necessity, and are not constrained by phylogeny.

**Conclusions/Significance:**

Ovipositor structure mirrored the increasingly complex trophic ecology and requirements for host accessibility in this parasite community. Ovipositor structure could be a useful surrogate for predicting the biology of parasites in other communities.

## Introduction

Resource partitioning and niche separation may require special adaptations which could be morphological, physiological, and/or behavioural [1]–[8]. The ovipositors of parasitic Hymenoptera have been critical in their exploitation of physical niches and host species for egg laying [9]–[14]. While host partitioning by Hymenoptera has been examined in several galler or parasitoid communities [15]–[17], this has rarely been examined for communities of both gallers and parasitoids that are parasitic on a single host plant species [18], [19]. Furthermore, the ovipositor adaptations that facilitate such partitioning of a single host plant species have not been considered, except for separation along the dimension of ovipositor length [13], [20]–[25]. Hymenoptera that parasitise hidden hosts buried within plant tissue also require tools and sensory structures on the ovipositor to cut into plant tissue and guide the ovipositor towards its target [12], [26]. This is especially important for those Hymenoptera whose first instar larvae are relatively immobile and do not engage in host-seeking behaviour [27], [28]. In such cases and especially with hidden hosts, eggs have to be precisely laid on/in suitable hosts using only the sensory guidance systems available on the ovipositor since first instar larvae cannot employ host-seeking behaviour as exhibited in some Hymenoptera, i.e. Perilampidae, Eucharitidae (Chalcidoidea) and Eucerotinae (Ichneumonidae) [27], [28]. Besides physical structure and the chemosensory apparatus, the degree of sclerotization of the ovipositor may also affect the stiffness of the ovipositor [29], [30] and may be important in providing access to hidden hosts protected by tough tissues. The structure and sclerotisation of the hymenopteran ovipositor as well as the arrangement of chemosensory or mechanosensory structures, i.e. sensilla, on it are critical for the utilization of the right host [10], [12], [26]. When the host plant is subject to parasitism by several hymenopteran parasites, inter-specific differences in all these ovipositor properties could facilitate partitioning within the single resource.

Knowledge of ovipositor structure and host-reaching capabilities especially in parasitic species is fundamental in determining enemy-free space for the host species which in turn has an impact on host and parasite community structure [31], [32]. Inter-specific differences in ovipositors may be exaggerated when the community of interacting hymenopterans is both highly host plant and prey specific, as well as closely related [13], [33]. Investigations of such communities would, therefore, also afford greater understanding of the extent of evolutionary lability versus conservatism in ovipositor structure depending on niche specificity. The community of wasps associated with fig plants is ideal for such an investigation. This community is characterized by high species specificity, close relatedness, and includes a variety of plant gallers as well as parasitoids/inquilines all of which develop within the same plant structure called the syconium which is a globular and enclosed inflorescence [34]–[36]. Furthermore, fig wasp larvae do not exhibit host-seeking behaviour; consequently egg deposition has to be precise. Also, the syconium is a physically and chemically crowded space [37] within which hundreds of wasps of the various species develop. Such a closed community would therefore afford a critical examination of how wasp species, in order to coexist, might need to differ in their ovipositors to locate and reach different hosts or host stages within the syconium, a question which is not so easily answered in open communities.

**Figure 1:**
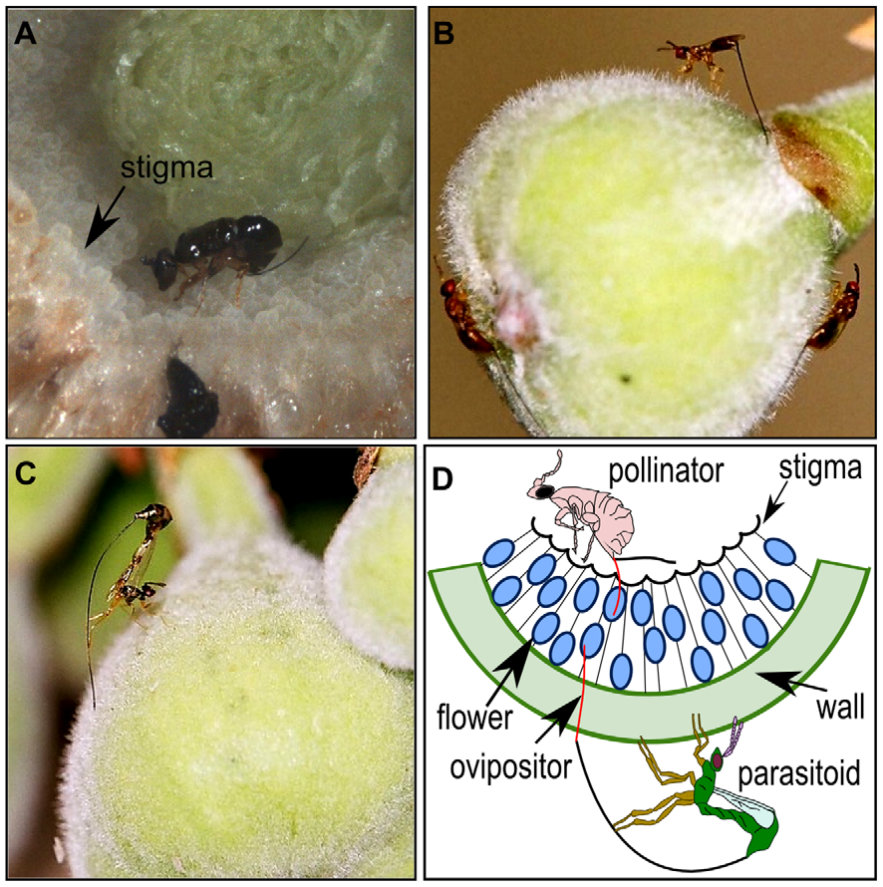
Oviposition strategies of fig wasps. Representative wasp species of the *Ficus racemosa* community demonstrating internal and external oviposition. (A) internally ovipositing pollinator *Ceratosolen fusciceps*, (B) externally ovipositing *Apocryptophagus testacea*, (C) externally ovipositing *Apocrypta* sp. 2 and (D) schema illustrating internal and external oviposition modes.

Fig wasps may oviposit by entering the syconium, or oviposition may occur from the outside using the ovipositor to penetrate the syconium (Fig. 1A–D). Pollinating fig wasps entering into the syconium insert their ovipositors into the style of female flowers to deposit their eggs [38] and here the target is not hidden as they can also have antennal and tarsal contact with the flowers. However, all wasps that oviposit from the outside of the syconium are seeking hidden hosts; the only way of assessing the suitability of a host for egg laying is through the ovipositor which has to drill through the fig wall to reach it. Here, we attempt to understand host location mechanisms and corresponding ovipositor adaptations using light and electron microscopy for the entire fig wasp community associated with a single *Ficus* species. We describe the morphology of the ovipositors including the types and distribution of their sensilla. We measure the degree of sclerotisation of each ovipositor and also the toughness of the figs across various stages of fig development. Wasps ovipositing into small/young and soft figs or those that enter into the syconia (i.e. pollinators) may be expected to have lesser sclerotisation of their ovipositor tips compared to those wasps ovipositing into larger/developmentally older and tougher figs. Also, wasps at a lower trophic level (e.g. early-arriving gallers) might be expected to have fewer types of ovipositor sensilla compared to wasps at a higher trophic level (e.g. later-arriving parasitoids) because the ovipositors of later-arriving wasps need to navigate through increasing chemical diversity as the syconium gets populated by various developing wasp species. Within this chemically diverse landscape, parasitoids need to find the appropriate development stage of their specific host insect species, possibly necessitating greater sensory precision in parasitoids compared to the early-arriving gallers that may only need to locate floral tissue. To the best of our knowledge, this is the first study on host resource partitioning via varied ovipositor tools in a closed parasitic community. We expected to find an increase in complexity of tools from internal to external ovipositing species and also from early ovipositing species to late arriving species.

## Materials and Methods

### Natural History of the Study System

The fig syconium (globular enclosed inflorescence) is the structure within which seeds are produced and wasps (Hymenoptera: Agaonidae) develop. In typical monoecious figs, pollinating female wasps enter the syconium through an opening called the ostiole, pollinate some female flowers resulting in seeds, and gall other flowers into each of which an egg is laid [38]. The pollinator offspring develop within the galled flowers. The non-pollinating fig wasps (NPFWs), i.e. parasites, usually do not enter the fig syconium but oviposit into the syconium from the outside, using long ovipositors [23] (Fig. 1). The parasites could be flower gallers, parasitoids, cleptoparasites or inquilines [25], [34], and arrive for oviposition into the syconium either before or after the pollinator in a specific sequence [6], [23], [39].

The fig wasp community of *Ficus racemosa* L. (Section: Sycomorus) from South India (Indian Institute of Science campus, Bangalore, Karnataka, 12°58′N and 77°35′E) was chosen for the study as this species has been a model system for investigations on resource partitioning via differences in life history and chemistry [6], [25], [37], [39], [40]. The community comprises one pollinating wasp species (*Ceratosolen fusciceps* Mayr: Agaonidae) and six species of NPFWs within the Sycophaginae (*Apocryptophagus*) and the Sycoryctinae (*Apocrypta*) (*Apocryptophagus stratheni* Joseph, *Apocryptophagus testacea* Mayr, *Apocryptophagus fusca* Girault, *Apocryptophagus agraensis* Joseph, *Apocrypta* sp. 2, *Apocrypta westwoodi* Grandi) [25]. All species differ in their timing of oviposition during syconium development, arriving either at the pre-pollination (A phase), pollination (B phase) or wasp development (C phase) periods but their offspring leave the syconium at the same time (D phase) when male pollinators cut an exit hole through the syconium wall. The wasps arrive in the following sequence for oviposition: *A. stratheni* (A phase), *A. testacea* (A phase), *C. fusciceps*+*A. fusca* (arrive concurrently in B phase), *Apocrypta* sp 2 (B and C phases), *A. agraensis* (C phase), and *Apocrypta westwoodi* (C phase) [25], [39]. *Ceratosolen fusciceps*, *A. stratheni*, *A. testacea* and *A. fusca* are gallers [6], [41], *A. agraensis* is most likely a parasitoid [25], [39], [41], while *Apocrypta* sp. 2 and *A. westwoodi* are definitely parasitoids [6], [41].

### Ovipositor Traits

While sclerotisation and melanization are independent processes [42], and both can contribute to the stiffening of biomaterials such as the ovipositor cuticle [43], the relative darkening of the ovipositor whether by sclerotisation and/or melanisation may be taken to indicate the relative extent of its stiffness [30]. The ovipositor consists of three valves; the upper valve and a pair of lower valves are connected by a joint and groove or olisthether mechanism [12], [26]. These together form the egg canal. The ovipositor sheath [12], [26] was not considered in this study since it does not enter plant tissue. The valves of each ovipositor were photographed using a light microscope (Zeiss Axioskop 2); photographs were taken up to a distance of 0.15 mm from the ovipositor tip since sclerotisation (i.e. darkening), if any, only occurred within this distance from the tip. Uniform lighting was maintained for all photography. Microscope images were imported to GIMP 2.6.8 (open access image manipulation software) and converted to grey scale pixels. Degree of darkening (intensity of sclerotisation) of the tip was quantified on an RGB scale (0 = absolute black, 255 = pure white). Thus a darker ovipositor tip would get a mean value closer to zero and a lighter one would be placed closer to 255. For measuring the percent area sclerotised, values between 0–254 were considered as being representative of sclerotisation, since a value of 255 indicates pure white. A non-parametric correlation, using Kendall's rank correlation test, was performed between the mean percent area of sclerotisation and the oviposition timing of each wasp species in terms of the first and last day (i.e. range) from the initiation of syconium development when the species was observed to be ovipositing (data from ref. [25]). A Kendall's rank correlation test was also performed between the oviposition timing of each wasp (as above) and the intensity of sclerotisation. We expected wasps ovipositing into softer syconia (e.g. A phase) to have less sclerotisation of their ovipositor tips compared to those ovipositing into harder syconia (e.g. C phase). We therefore measured the force required to penetrate the wall of A-, B- and C-phase syconia. Since the C phase is of long duration, we specifically measured penetration forces for syconia in early C phase at the time when *Apocrypta* sp 2, the most common parasitoid (Ghara & Borges, unpublished data), was observed ovipositing into the figs. For the penetration measurements we used a standard entomological pin (Asta Ento Pins, 38×0.45 mm, Newey Goodman Ltd. England) and measured the force (in newtons) required by the pin (moving at a constant speed of 0.05 mm s^−1^) to penetrate syconia of A, B and C phases (*N* = 6 in each phase) using an INSTRON 5567 force testing instrument. For these measurements, figs in A and B phases were obtained from one tree and those in C phase from another tree; thus there was no inter-tree variation for measurements within a phase.

Scanning electron microscopy (SEM) was used to elucidate the ultrastructure of the wasp ovipositors. Desiccated ovipositors were sputter-coated with gold and viewed in a FEI Quanta 200 ESEM. Measurements of various ovipositor features including sensilla dimensions were made using ImageJ software (version1.40 g) from these SEM images. We have designated sensilla to types based on the literature available on sensilla morphology (e.g. [44], [45], [46]). The distance of each type of sensillum from the ovipositor tip was also measured.

### Assembling the Fig Wasp Community Using the Tools of the Ovipositor Trade

We used the morphological features of the ovipositor such as the area sclerotised, intensity of sclerotisation, presence of teeth on the upper and lower valves, and types of sensilla to perform a hierarchical cluster analysis using Euclidean distance and Ward's method. Ward's method uses ANOVA to evaluate distance between clusters and is considered to be very efficient, especially since it can determine clusters of small sizes, and thus at finer scales [25]. We provide the approximately unbiased (AU) as well as bootstrapping (BP) values for these clusters. The data matrix used binary characters for teeth on the valves and types of sensilla, and continuous characters for percent and intensity of sclerotisation. Clusters were built in R (version 2.11.1) (R Development Core Team 2009) [47] using the *pvclust* algorithm to determine whether ovipositor traits mirror what is already known about the ecology of the members of this fig wasp community.

## Results

### Ovipositor Sclerotisation

**Figure 2:**
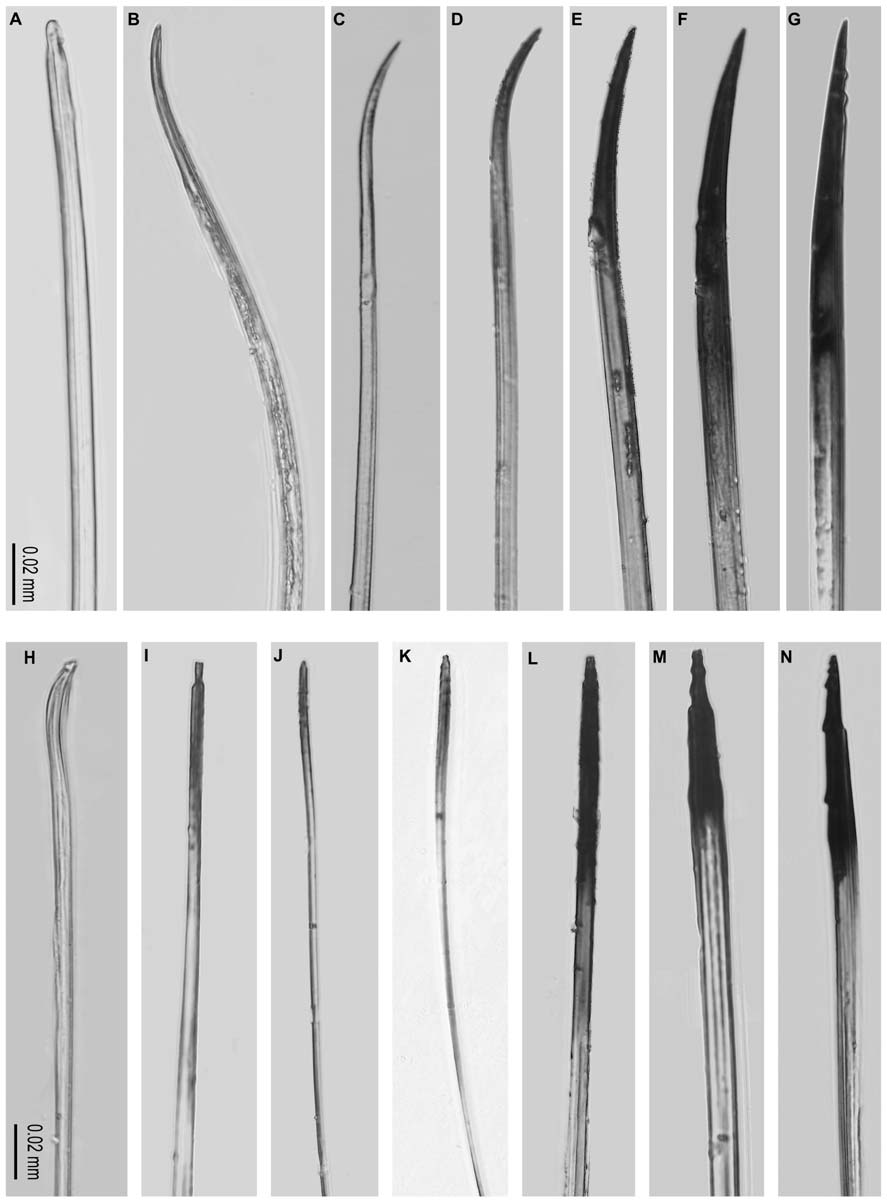
Ovipositor sclerotisation using light microscopy. Ovipositor sclerotisation (A–G) lower valve, (H–N) upper valve. (A, H) Pollinator *C. fusciceps*, (B, I) *A. stratheni*, (C, J) *A. testacea*, (D, K) *A. fusca*, (E, L) *A. agraensis*, (F, M) *Apocrypta* sp. 2, (G, N) *Apocrypta westwoodi*. Scale = 0.02 mm in all cases.

**Figure 3:**
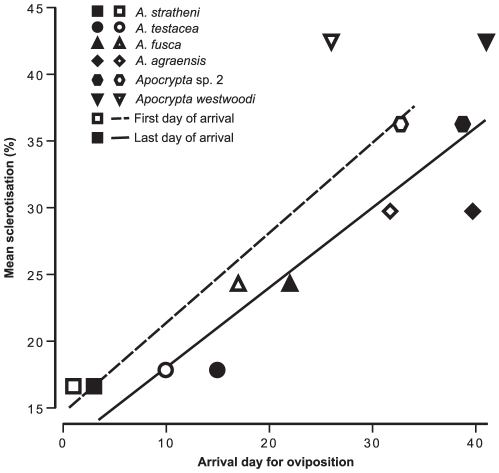
Relatioship between ovipositor sclerotisation and wasp arrival time. Relationship between mean percent area of sclerotisation and oviposition sequence for externally ovipositing wasp species. Open symbols and dashed line indicate first day of arrival of each wasp species for oviposition; closed symbols and solid line indicate last day of arrival of each wasp species for oviposition. The x-axis depicts temporal progression in syconium development.

**Figure 4:**
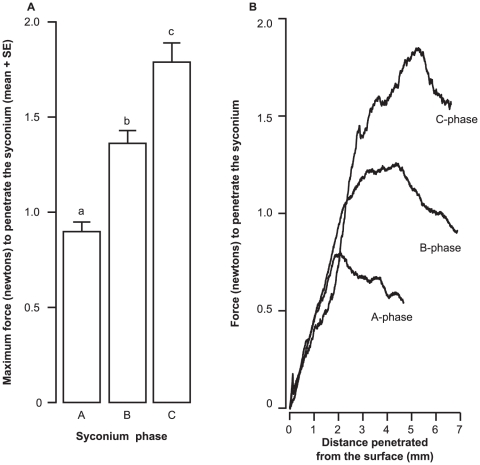
Strength of the fig syconium estimated through needle penetration study. (A) Maximum force (newtons) required to penetrate the syconium (A–C phases), and (B) a representative data sample of force required to penetrate the syconium with increasing distance from the syconium exterior (A–C phases).

The degree of ovipositor sclerotisation increased from the early-arriving gallers to the later-arriving parasitoids with negligible sclerotisation (i.e. darkening) observed in the pollinator ovipositor (Fig. 2). Percent area of sclerotisation and first day of arrival for oviposition by each wasp species were significantly related (Fig. 3; Kendall's *τ* = 0.73, *P* = 0.05, *N* = 6) as also the last day of arrival of each wasp species (Fig. 3; Kendall's *τ* = 0.86, *P* = 0.01, *N* = 6), indicating that the ovipositors of later-arriving wasps were sclerotised over greater areas. The pollinator was excluded from this analysis since it oviposits internally and it had negligible sclerotisation. There was a trend (although not significant) towards greater intensity of sclerotisation (i.e. darkening) of the ovipositor with both first (Kendall's *τ* = −0.46, *P* = 0.27, *N* = 6) and last day of arrival of each wasp species (Kendall's *τ* = −0.6, *P* = 0.13, *N* = 6) (*τ* values are negative since 0 in RGB = black; any value closer to zero indicates greater darkening). We found a significant difference between the maximum force required to penetrate the syconium from A to C phases (Fig. 4A; Mann-Whitney comparisons: A versus B phases: *U* = 1, *P* = 0.006, *N* = 12; B versus C phases: *U* = 0, *P* = 0.004, *N* = 12; A versus C phases: *U* = 0, *P* = 0.004, *N* = 12); therefore the hardness of syconia increased from A to C phases (Figs. 4A, B). In all phases the required penetration force was seen to decline after 2–4 mm of penetration distance (Fig. 4B) indicating that ovipositors (especially their tips) need to overcome considerable initial resistance to penetration. Thus, the increase in sclerotisation of the ovipositor tips with the lateness of arrival of the wasps for oviposition matched the increasing hardness of syconia from A to C phases.

### Ovipositor Ultrastructure and the Fig Wasp Community

**Figure 5:**
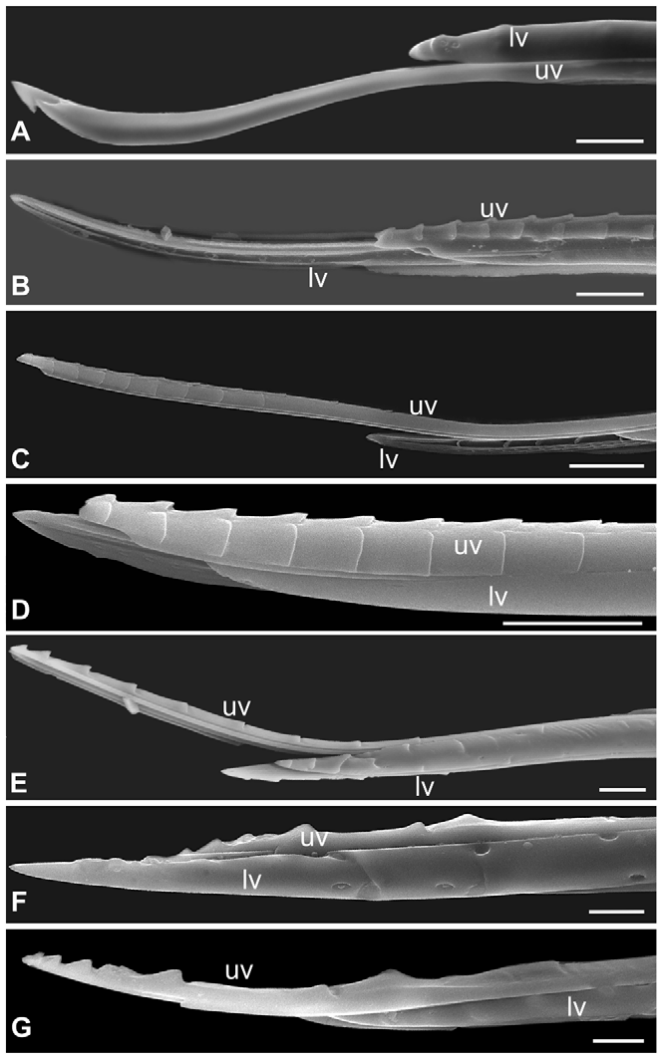
Scanning electron micrographs (SEMs) showing ovipositor morphology. (A) Pollinator *C. fusciceps*, (B) *A. stratheni*, (C) *A. testacea*, (D) *A. fusca*, (E) *A. agraensis*, (F) *Apocrypta* sp. 2 and (G) *Apocrypta westwoodi*; uv = upper valve, lv = lower valve. Scale = 10 µm.

The internally-ovipositing pollinator was the only wasp that had only a single notched tooth on the upper ovipositor valve while all externally-ovipositing NPFWs (gallers and parasitoids) had multiple teeth on the upper valve (Table S1, Fig. 5). All gallers were characterised by the absence of teeth on the lower ovipositor valves (pollinator and NPFW gallers) while all parasitoids had teeth on the lower valves (Table S1) including *Apocryptophagus agraensis*. Teeth height increased in the parasitoids relative to the gallers (Fig. 5). The number and types of sensilla increased from the early-arriving gallers to the later-arriving parasitoids (Tables S1 and S2). The ovipositor of the internally ovipositing *C. fusciceps* had only a single type of sensillum that occurred in a triad on each lower valve (Fig. S1). All galling NPFWs in the genus *Apocryptophagus* had various types of campaniform sensilla and several unidentified sensillum types (Table S1, Fig. S1). The putative parasitoid *Apocryptophagus agraensis* had only basiconic sensilla besides an unidentified type (Table S1, Fig. S1). The diversity of sensilla was highest in the *Apocrypta* parasitoids with basiconic, campaniform, coeloconic as well as several unidentified sensilla types (Table S1, Fig. S1). Most sensilla were found concentrated at the tip of the ovipositors (Table S2); however, campaniform and coeloconic sensilla in the *Apocrypta* parasitoids were also found farther away from the tip (e.g. up to 174 µm from the tip in campaniform sensillum type 4 (CS4) in *A. westwoodi*; Table S2).

### Assembling the Fig Wasp Community Using the Tools of the Ovipositor Trade

**Figure 6:**
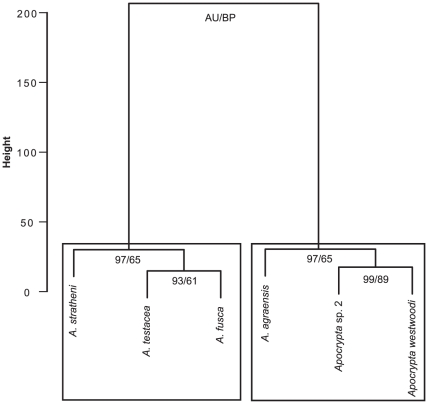
Clustering of fig wasps based on ovipositor characters. Clustering was done using Ward's method. AU = approximately unbiased (AU) and BP = bootstrap values.

Ovipositor traits generated clusters with uniformly high approximately unbiased (AU) values (Fig. 6) indicating good resolution of functional groupings (i.e. gallers versus parasitoids); the BP values were not as high. AU values are considered better than BP values as indicators of the stability of clusters since the AU method involves multi-scale bootstrapping [47]. All *Apocryptophagus* gallers were grouped into one cluster with a second cluster that included the two *Apocrypta* parasitoids along with the putative parasitoid *Apocryptophagus agraensis* (Fig. 6). While these clusters were performed without including the galler pollinator, the results of the clustering relationships did not change when the pollinator was included (results not shown).

## Discussion

The gross morphology as well as the ultrastructure of the ovipositors of the wasps of *F. racemosa* showed adaptations that mirrored their ecology and mode of oviposition. The internally ovipositing galler pollinator had the simplest ovipositor with negligible sclerotisation and only a single type of sensillum. The degree of ovipositor sclerotisation increased with the timing of wasp species arrival for oviposition into the syconium, reflecting the greater resistance to oviposition faced by the later-arriving externally ovipositing fig wasps that oviposit into harder syconia. In general, gallers had fewer types of sensilla compared to the parasitoids. The ovipositor of *Apocryptophagus agraensis* was different from that of congeneric and syntopic galler species and clustered with the *Apocrypta* parasitoids reflecting its parasitoid ecology, and thus the evolutionary lability of certain ovipositor traits based on adaptive necessity.

Fig syconia undergo a change from early stage soft figs to later stage hard figs (A–C phases) (Fig. 4). The differential sclerotisation of the ovipositor tip indicates that the wasps require greater sclerotisation and therefore stiffer ovipositor tips for penetrating the fig wall at later development stages of the syconium. Ovipositor length also correlates with the arrival sequence of the wasps [25], and syconium wall thickness (Ghara & Borges, unpublished data) [48]. While the ovipositors of all the NPFWs which oviposit externally were found to have teeth, the depth of these serrations differed qualitatively with the lateness of arrival for oviposition indicating the need for adaptations to cut through harder and thicker syconia. These teeth are also mineralised (Kundanati *et al.*, unpublished data). However, only the ovipositors of parasitoids (including *A. agraensis*) had teeth on both upper and lower valves. Since gallers arrive when the figs are softer (A and B phases), completely toothed ovipositors may only be needed at later stages. Furthermore, the two *Apocrypta* parasitoids oviposit by arching their abdomen as well as their extra-long ovipositors while the *Apocryptophagus* gallers oviposit while lying flat on the fig surface (Fig. 1). However, the biomechanics of the drilling process is barely understood [10], [14]. The drilling process can be extended, e.g. between 25–55 min in *Apocrypta westwoodi* [48]. In strict contrast, the internally ovipositing pollinator, which has merely to insert its ovipositor down the style of the flower, has only a pre-apical notch and no serrations on its ovipositor. In general, serrations aiding the drilling process are less well developed in parasitoids whose hosts are exposed [49].

The diversity of sensilla increased with the lateness of arrival of the fig wasps for oviposition as well as with the mode of parasitism. Parasitoids had the most types of sensilla compared to gallers with the exception of the parasitoid *A. agraensis* that had only two types of sensilla. The internally ovipositing pollinating wasp had only a single sensillum type. Since pollinating female fig wasps lose their distal antennal segments in the process of entering the fig through the ostiole [34], [50], the pollinator female may find suitable oviposition sites using chemoreceptors on basal antennal segments, tarsi and the few sensilla (three only) on the ovipositor tip. Although these appear to be campaniform sensilla and therefore typically cuticular strain detectors [45], [51], they may also have a dual mechano-chemoreceptor function as in the braconid parasitic wasp *Orgilus lepidus* [52]. Sensilla present on pilose structures on the antennae or on the ovipositors of ectoparasites targeting exposed hosts may not require specific adaptations for protection from damage; however the ovipositors of fig wasps, especially those that pierce the fig wall for oviposition can only have shielded sensilla (i.e. receptors within pits) to avoid contact damage while penetrating plant tissue. The potential damage to sensilla during syconium penetration can also be exacerbated by sticky latex which is characteristic of plants in the Moraceae, such as *Ficus* [53]. Therefore it is probably more likely that many pit-like sensilla on such ovipositors will have a dual mechano-chemoreceptor function, although this is not yet known. In our study, most sensilla, especially the largely chemosensory basiconic and coeloconic types, were concentrated at the tips of the ovipositors. In the parasitoids, campaniform sensilla were also found at considerable distance from the tip, and since these sensilla are largely mechanoreceptors, it could be adaptive for them to be spread over larger portions of the ovipositor as found in many parasitoid taxa [54]. This will enable them to sense stresses and strains offered by different types of tissues along their entire lengths and could help in locating the right target. In ectoparasitoids, where the ovipositor tip needs to examine host surfaces, the sensilla are also concentrated at the tip [11]. In all fig wasp species, sensilla were present only on the lower valves as has been observed in other parasitic wasps [55]. Similarly many ichneumonids which are endoparasitoids of concealed hosts have no sensilla on the upper valve; the upper valve is used for supporting the lower valves and in interlocking with the substrate during penetration, while the lower valves are involved in venom injection and bear chemoreceptors at the tip [12].

Steering mechanisms and the extent of ovipositor flexibility are still not known and have been scarcely studied in parasitic wasps [12], [26]. The tortuous paths taken by fig wasp ovipositors inside the syconium (Fig. S2), and their extended oviposition periods, could indicate that these wasps even lay a clutch of eggs inside a syconium before pulling out the ovipositor to lay eggs in the same or different fig syconia; this may occur in the early-arriving gallers *A. stratheni* and *A. testacea* whose offspring generally develop in clusters of galls or galled flowers (M. Ghara, personal observation). An additional ovipositor requirement is ease of ovipositor removal, and some wasps, while ovipositing, face predation pressure from ants [39], being attacked while their ovipositors are inserted into the syconium. However, ovipositor removal mechanisms have not been studied in parasitic wasps. Most importantly, while in many plant-galler communities, gall morphology itself serves to afford protection to gallers from parasitoids via the construction of “enemy-free space” [31], parasites of fig wasps acquire access to their hosts by penetrating through the barrier of the fig syconium wall, which offers considerable initial resistance as we have shown (Fig. 4B). This in turn may have led to the low diversity and complexity of fig wasp galls [22] necessitating specific ovipositor adaptations to cut through the syconium wall, and flexible ovipositors that pass through several galls, if necessary, in seeking the right host.

The clustering of the fig wasp community members of *F. racemosa* using ovipositor features was also concordant with the clustering obtained using various life-history traits of the same community where *Apocryptophagus agraensis* which belongs to the “galler” genus *Apocryptophagus* was also found to cluster with the parasitoid genus *Apocrypta* [25]. Yet, one conserved life-history trait in *Apocryptophagus agraensis* was its pro-ovigeny (i.e. all eggs are mature at eclosion) which was similar to its congeners; on the other had, parasitoids of the genus *Apocrypta* were synovigenic (i.e. progressively maturing their eggs after eclosion) which is typical of the parasitoid lifestyle [25]. Several other lines of independent evidence including the congruency between cuticular hydrocarbons of hosts and parasites (Ranganathan & Borges, unpublished data) indicate that *A. agraensis* is a parasitoid, more specifically a parasitoid of the pollinator *C. fusciceps*, as earlier speculated [25]. This concordance between life history, chemistry, ecology and now ovipositor morphology suggests that ovipositor traits can also be important in understanding the functional ecology of wasp communities. Moreover, the more parasitoid-like features of the ovipositor of *A. agraensis* compared to its syntopic galler congeners indicates that those features of the ovipositor that are evolutionary labile must be viewed with caution when used in a phylogenetic study since they could be subject to considerable homoplasy [56], based on adaptive necessity. Studies in section *Sycomorus*, to which *F. racemosa* belongs, indicate that pollinators and fig host species show cophylogeny whereas NPFWs, particularly parasitoids, show host shifts or more generalisation with a single parasitoid species parasitising several fig hosts or fig wasps within a single host [57], [58]. While more recent evidence is accumulating that single fig wasp pollinator species may also pollinate several fig species [59] and that the one fig-one pollinator rule may have exceptions [60], this may only be possible if the ostiole (entrance into the syconium) and chemical barriers can be breached by the pollinators; a similar argument may be advanced for the NPFWs. Molecular phylogeny studies have indicated that congeneric fig wasp species attacking the same host but differing in ovipositor length and oviposition time are likely to be sister species [33]. *Apocryptophagus* species associated with *F. racemosa* might therefore be sister species as they exhibit differences in ovipositor length and oviposition timing [25], [39]; however they could also be an assemblage of related species which have switched hosts. Only phylogenetic studies using both morphology and gene sequences may help to resolve these issues. The similarities seen in several features of the ovipositor in members of the genus *Apocryptophagus* could, therefore, also indicate phylogenetic conservatism of such traits. Ovipositor morphology of fig wasps can also be a constraint selecting for innovative behavioural adjustments, such as those in African *Watshamiella* which are reported to have “delicate” ovipositors and to use the holes drilled into fig syconia by *Apocrypta* parasitoids for oviposition [61]. Clearly ovipositor structure is an important axis for niche separation in parasitic hymenopteran communities and deserves much more attention. The generality of oviposition syndromes in terms of ovipositor morphology should also be tested across different wasp communities across different hosts.

In conclusion this paper has demonstrated the following important general points. 1) Ovipositor structure can mirror the increasingly complex ecology of coexisting species in a host–parasite community and can contribute to niche separation; 2) Some ovipositor tools show lability based on adaptive necessity, and are not constrained by phylogeny; and 3) Ovipositor structure could be a useful surrogate for predicting the biology of members of other parasitic communities.

## Supporting Information

Figure S1 **Scanning electron micrographs (SEMs) of sensilla.** The various types of sensilla observed on the lower valve of the ovipositors as indicated by the arrows. (A–E) CS 1–5 = campaniform sensilla, (F–G) BS = basiconic sensilla, (H) CoS = coeloconic sensilla, (I–N) UN 1–6 = unidentified. Scale = 2 µm.
(TIF)

Figure S2 **Ovipositor movement into the syconium in fig wasps of** ***Ficus racemosa*** **.** (A) Ovipositor passing through the wall with the fig wasp lying flat on the fig surface for oviposition and (B) Ovipositor navigating through flowers. The path of the ovipositor is indicated by arrows.
(TIF)

Table S1 **Morphological traits of the ovipositors of the fig wasps of** ***F. racemosa*** **.**
(DOC)

Table S2 **Dimensions of sensilla observed on the lower valve of ovipositors of fig wasps of** ***F. racemosa*** **.**
(DOC)

## References

[1] Donaldson JS (1992). Adaptation for oviposition into concealed cycad ovules in the cycad weevils *Antliarhinus zamiae* and *A. signatus* (Coleoptera: Curculionoidea).. Biol J Linn Soc.

[2] Rohde K (1994). Niche restriction in parasites: proximate and ultimate causes.. Parasitology.

[3] Albrecht M, Gotelli NJ (2001). Spatial and temporal niche partitioning in grassland ants.. Oecologia.

[4] Abzhanov A, Protas M, Grant BR, Grant PR, Tabin CJ (2004). *Bmp4* and morphological variation of beaks in Darwin's finches.. Science.

[5] Siemers BM, Swift SM (2006). Differences in sensory ecology contribute to resource partitioning in the bats *Myotis bechsteinii* and *Myotis nattereri* (Chiroptera: Vespertilionidae).. Behav Ecol Sociobiol.

[6] Proffit M, Schatz B, Borges RM, Hossaert-McKey M (2007). Chemical mediation and niche partitioning in non-pollinating fig-wasp communities.. J Anim Ecol.

[7] Smadja C, Butlin RK (2009). On the scent of speciation: the chemosensory system and its role in premating isolation.. Heredity.

[8] Wittman SE, Sanders NJ, Ellison AM, Jules ES, Ratchford JS (2010). Species interactions and thermal constraints on ant community structure.. Oikos.

[9] Field SA, Austin AD (1994). Anatomy and mechanics of the telescopic ovipositor system of *Scelio* Latrielle (Hymenoptera: Scelionidae) and related genera.. Int J Insect Morphol Embryol.

[10] Quicke DLJ, Fitton MG, Tunstead JR, Ingram SN, Gaitens PV (1994). Ovipositor structure and relationships within the Hymenoptera with special reference to the Ichneumonoidea.. J Nat Hist.

[11] LeRalec AA, Rabasse JM, Wainberg E (1996). Comparative morphology of the ovipositor of some parasitic Hymenoptera in relation to the characteristics of their hosts.. Can Entomol.

[12] Quicke DLJ, LeRalec A, Vilhelmsen L (1999). Ovipositor structure and function in the parasitic Hymenoptera with an exploration of new hypotheses.. Entomol Rendiconti.

[13] Weiblen GD (2004). Correlated evolution in fig pollination.. Syst Biol.

[14] Vilhelmsen L, Turrisi GF (2011). Per arborem ad astra: Morphological adaptations to exploiting the woody habitat in the early evolution of Hymenoptera.. Arthropod Struct Dev.

[15] Price PW (1972). Parasitoids utilizing the same host: adaptive nature of differences in size and form.. Ecology.

[16] Sanver D, Hawkins BA (2000). Galls as habitats: the inquiline communities of insect galls.. Basic Appl Ecol.

[17] Cook JM, Rokas A, Pagel M, Stone GN (2002). Evolutionary shifts between host oak sections and host-plant organs in *Andricus* gallwasps.. Evolution.

[18] Jousselin E, van Noort S, Berry V, Rasplus J-Y, Rønsted N (2008). One fig to bind them all: host conservatism in a fig wasp community unraveled by cospeciation analyses among pollinating and nonpollinating fig wasps.. Evolution.

[19] Bailey R, Schönrogge K, Cook JM, Melika G, Csóka G (2009). Host niches and defensive extended phenotypes structure parasitoid wasp communities.. PLoS Biol.

[20] Brandl R, Vidal S (1987). Ovipositor length in parasitoids and tentiform leaf mines: adaptations in Eulophids (Hymenoptera: Chalcidoidea).. Biol J Linn Soc.

[21] Bronstein JL (1991). The nonpollinating wasp fauna of *Ficus pertusa*: exploitation of a mutualism?. Oikos.

[22] Compton SG, Rasplus J-Y, Ware AB, Hawkins BA, Sheehan W (1994). African fig wasp parasitoid communities.. Parasitoid community ecology.

[23] Kerdelhué C, Rasplus J-Y (1996). Non-pollinating Afrotropical fig wasps affect fig–pollinator mutualism in *Ficus* within the subgenus *Sycomorus*.. Oikos.

[24] Sivinski J, Vulinec K, Aluja M (2001). Ovipositor length in a guild of parasitoids (Hymenoptera: Braconidae) attacking *Anastrepha* spp fruit flies (Diptera: Tephritidae) in southern Mexico.. Ann Entomol Soc Am.

[25] Ghara M, Borges RM (2010). Comparative life-history traits in a fig wasp community: implications for community structure.. Ecol Entomol.

[26] Quicke DLJ, Fitton MG (1995). Ovipositor steering mechanisms in parasitic wasps of the families Gasteruptiidae and Aulacidae (Hymenoptera).. Proc R Soc B.

[27] Eggleton P, Belshaw R (1993). Comparison of dipteran hymenopteran and coleopteran parasitoids: provisional phylogenetic explanations.. Biol J Linn Soc.

[28] Brodeur J, Boivin G (2004). Functional ecology of immature parasitoids.. Annu Rev Entomol.

[29] Vincent JFV, Hillerton JE (1979). The tanning of insect cuticle—a critical review and a revised mechanism.. J Insect Physiol.

[30] Rouquette J, Davis AJ (2003). *Drosophila* species (Diptera: Drosophilidae) oviposition patterns on fungi: The effect of allospecifics, substrate toughness, ovipositor structure and degree of specialization.. Eur J Entomol.

[31] Stone GN, Schönrogge K (2003). The adaptive significance of insect gall morphology.. Trends Ecol Evol.

[32] Nyman T (2010). To speciate or not to speciate? Resource heterogeneity, the subjectivity of similarity, and the macroevolutionary consequences of niche-width shifts in plant-feeding insects.. Biol Rev.

[33] Weiblen GD, Bush GL (2002). Speciation in fig pollinators and parasites.. Mol Ecol.

[34] Cook JM, Rasplus J-Y (2003). Mutualists with attitude: coevolving fig wasps and figs.. Trends Ecol Evol.

[35] Herre EA, Jandér KC, Machado CA (2008). Evolutionary ecology of figs and their associates: recent progress and outstanding puzzles.. Annu Rev Ecol Evol Syst.

[36] Cruad A, Jabbour-Zahab R, Genson G, Couloux A, Peng Y-H (2010). Out of Australia and back: the world-wide historical biogeography of non-pollinating fig wasps (Hymenoptera: Sycophaginae).. J Biogeogr.

[37] Krishnan A, Muralidharan S, Sharma L, Borges RM (2010). A hitchhiker's guide to a crowded syconium: how do fig nematodes find the right ride?. Func Ecol.

[38] Galil J, Eisikowitch D (1968). Flowering cycles and fruit types of *Ficus sycomorus* in Israel.. New Phytol.

[39] Ranganathan Y, Ghara M, Borges RM (2010). Temporal associations in fig–wasp–ant interactions: diel and phenological patterns.. Entomol Exp Appl.

[40] Ranganathan Y, Borges RM (2009). Predatory and trophobiont-tending ants respond differently to fig and fig wasp volatiles.. Anim Behav.

[41] Wang R-W, Zheng Q (2008). Structure of a fig wasp community: temporal segregation of oviposition and larval diets.. Symbiosis.

[42] Andersen SO (2010). Insect cuticular sclerotization: A review.. Insect Biochem Mol Biol.

[43] Moses DN, Harreld JH, Stucky GD, Waite JH (2006). Melanin and *Glycera* jaws. Emerging dark side of a robust biocomposite structure.. J Biol Chem.

[44] Slifer EH (1970). The structure of arthropod chemoreceptors.. Annu Rev Entomol.

[45] Grünert U, Gnatzy W (1987). Campaniform sensilla of *Calliphora vicina* (Insecta Diptera).. Zoomorphology.

[46] Chapman RF (1998). The insects: structure and function.

[47] R Development Core Team (2009). R: A language and environment for statistical computing.

[48] Zhen WQ, Huang DW, Xiao JH, Yang DR, Zhu CD (2005). Ovipositor length of three *Apocrypta* species: effect on oviposition behavior and correlation with syconial thickness.. Phytoparasitica.

[49] Quicke DLJ (1997). Parasitic wasps.

[50] Kjellberg F, Jousselin E, Hossaert-McKey M, Rasplus J-Y, Raman A, Schaefer CW, Withers TM (2005). Biology, ecology and evolution of fig-pollinating wasps (Chalcidoidea, Agaonidae).. (2005) Biology, ecology and evolution of gall-inducing arthropods.

[51] Keil TA (1997). Functional morphology of insect mechanoreceptors.. Microsc Res Techniq.

[52] Hawke SD, Farley RD, Greany PD (1973). The fine structure of sense organs in the ovipositor of the parasitic wasp *Orgilus lepidus* Muesebeck.. Tissue Cell.

[53] Farrell BD, Dussourd DE, Mitter C (1991). Escalation of plant defense: Do latex and resin canals spur plant diversification.. Am Nat.

[54] van Veen JC (1981). The biology of *Poecilostictus cothurnatus* (Hymenoptera Ichneumonidae) an endoparasite of *Bupalus pinarius* (Lepidoptera Geometridae).. Ann Entomol Fenn.

[55] Gerling D, Quicke DLJ, Orion T (1998). Oviposition mechanisms in the whitefly parasitoids *Encarsia transvena* and *Eretmocerus mundus*.. Biocontrol.

[56] Laurenne N, Karatolos N, Quicke DLJ (2009). Hammering homoplasy: multiple gains and losses of vibrational sounding in cryptine wasps (Insecta: Hymenoptera: Ichneumonidae).. Biol J Linn Soc.

[57] Silvieus SI, Clement WL, Weiblen GD, Tilmon KJ (2007). Cophylogeny of figs, pollinators, gallers and parasitoids.. Specialization, speciation, and radiation: The evolutionary biology of herbivorous insects.

[58] McLeish MJ, van Noort S, Tolley KA (2010). African parasitoid fig wasp diversification is a function of *Ficus* species ranges.. Mol Phylogenet Evol.

[59] Compton SG, Grehan K, van Noort S (2009a). A fig crop pollinated by three or more species of agaonid fig wasps.. Afr Entomol.

[60] Lin R-C, Yeung CK-L, Fong JJ, Tzeng H-Y, Li S-H (2011). The lack of pollinator specificity in a dioecious fig tree: sympatric fig-pollinating wasps of *Ficus septica* in southern Taiwan.. Biotropica.

[61] Compton SG, van Noort S, McLeish M, Deeble M, Stone V (2009b). Sneaky African fig wasps that oviposit through holes drilled by other species.. Afr Nat Hist.

