# Supplementary figures and images for: Nature's Swiss Army Knives: Ovipositor Structure Mirrors Ecology in a Multitrophic Fig Wasp Community
Source: PLoS One. 2011 Aug 31;6(8):e23642. doi: 10.1371/journal.pone.0023642 (PMC3166121; doi:10.1371/journal.pone.0023642)

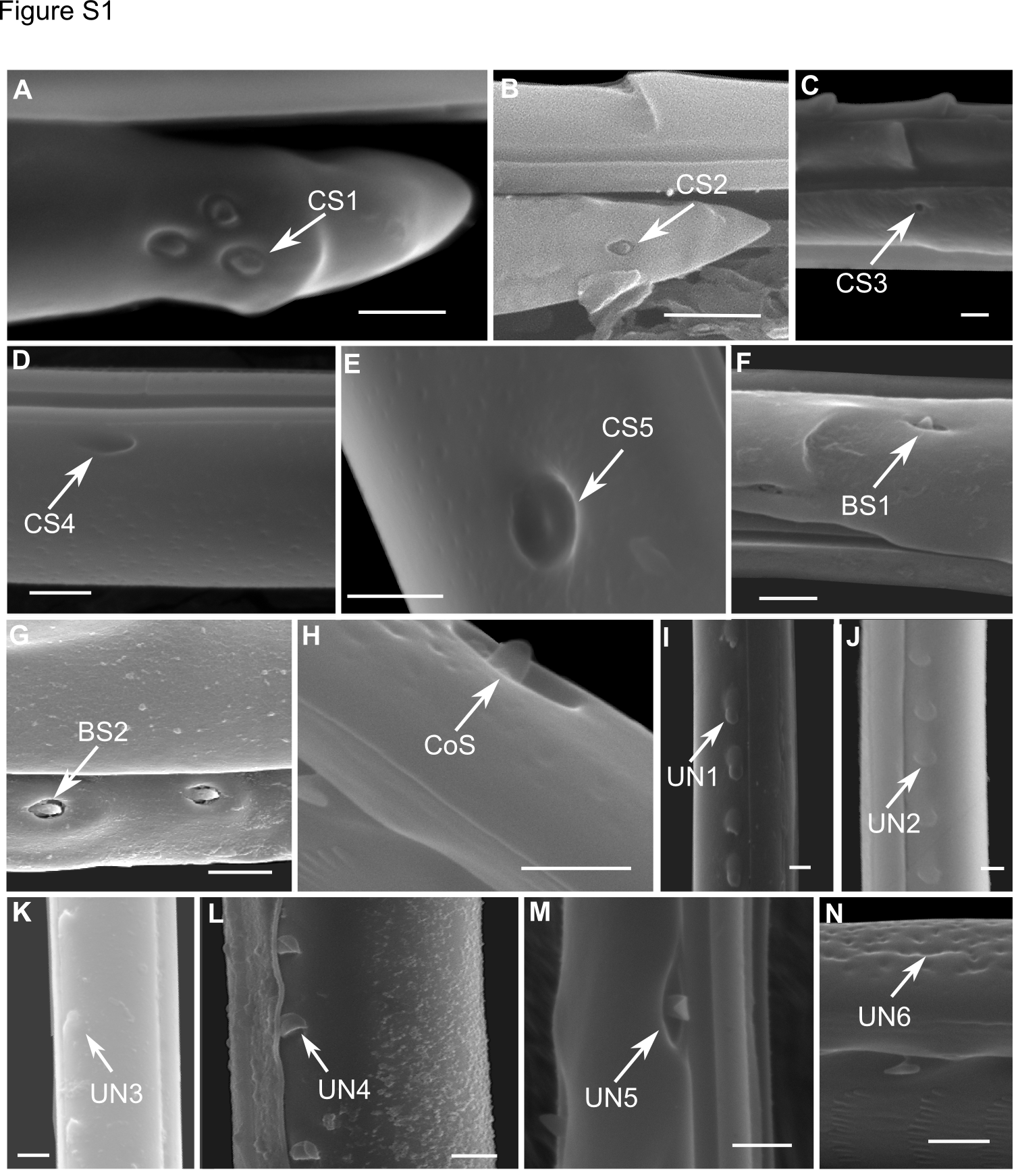

Supplement: Figure S1 — Scanning electron micrographs (SEMs) of sensilla. The various types of sensilla observed on the lower valve of the ovipositors as indicated by the arrows. (A–E) CS 1–5 = campaniform sensilla, (F–G) BS = basiconic sensilla, (H) CoS = coeloconic sensilla, (I–N) UN 1–6 = unidentified. Scale = 2 µm. (TIF) [file pone.0023642.s001.tif]

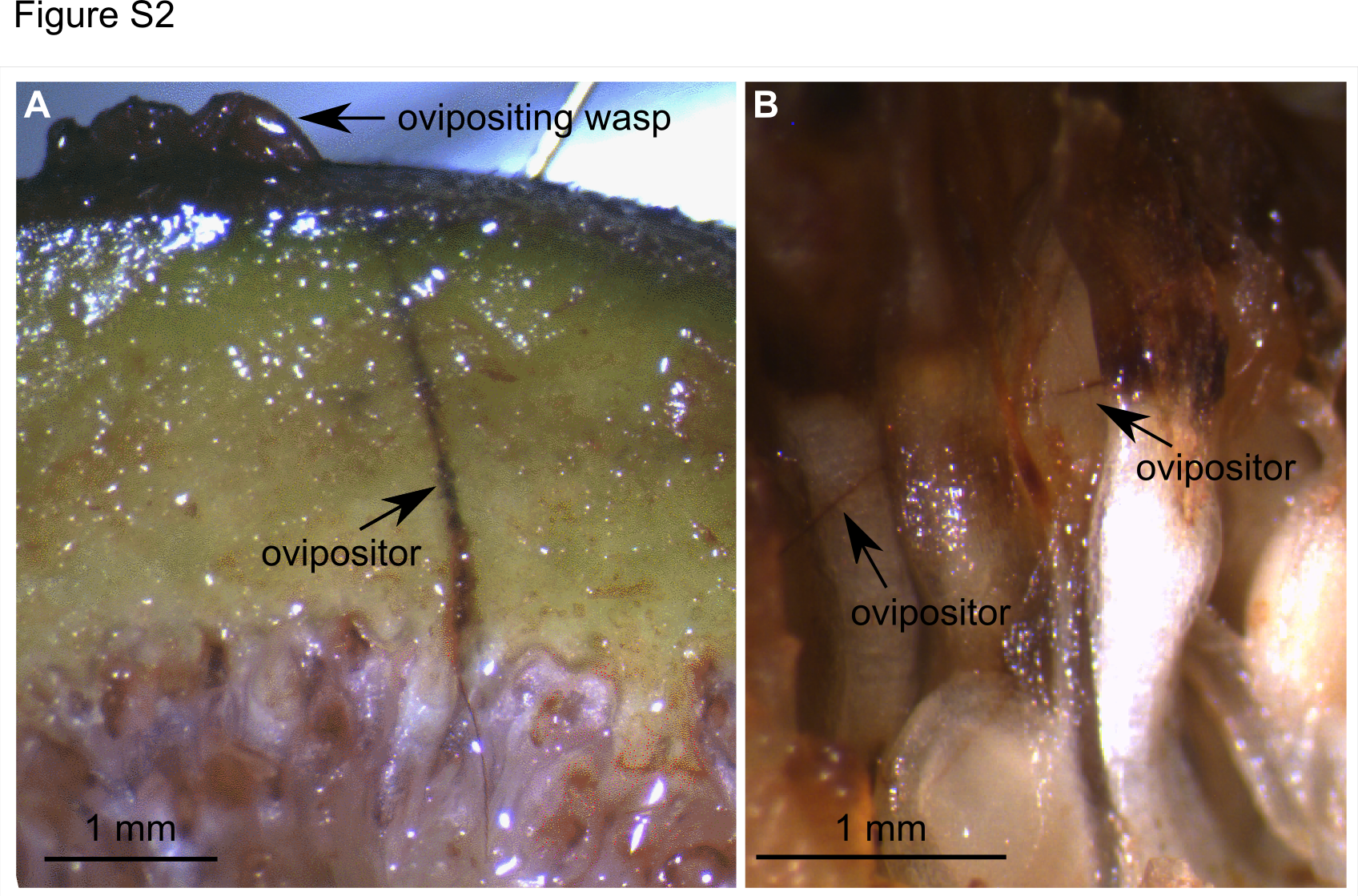

Supplement: Figure S2 — Ovipositor movement into the syconium in fig wasps of Ficus racemosa . (A) Ovipositor passing through the wall with the fig wasp lying flat on the fig surface for oviposition and (B) Ovipositor navigating through flowers. The path of the ovipositor is indicated by arrows. (TIF) [file pone.0023642.s002.tif]
