# Supplementary material for: Nature's Swiss Army Knives: Ovipositor Structure Mirrors Ecology in a Multitrophic Fig Wasp Community
Source: PLoS One. 2011 Aug 31;6(8):e23642. doi: 10.1371/journal.pone.0023642 (PMC3166121; doi:10.1371/journal.pone.0023642)
Supplement: Table S1 — Morphological traits of the ovipositors of the fig wasps of F. racemosa . (DOC) [file pone.0023642.s003.doc]

**Table S1.** Morphological traits of the ovipositors of the fig wasps of *F. racemosa*.

|  | ***C. fusciceps*** | ***A. stratheni*** | ***A. testacea*** | ***A. fusca*** |
| --- | --- | --- | --- | --- |
| **Parasitism mode/Type** | Galler/Pollinator | Galler/NPFW | Galler/NPFW | Galler/NPFW |
| **Teeth–upper valve** | Single pre-apical tooth | Multiple teeth | Multiple teeth | Multiple teeth |
| **Teeth–lower valves** | Absent | Absent | Absent | Absent |
| **Type of teeth** | Single pre-apical tooth | Moderate serrations | Moderate serrations | Moderate serrations |
| **Tip shape–upper valve** | Pointed | Notch | Notch | Notch |
| **Tip shape–lower valves** | Blunt | Pointed | Pointed | Pointed |
| **Tip sclerotisation (%) Mean ± SD** **(range) [n]** | 0 | 16.61 ± 3.88  (13.48–22.82) [5] | 17.83 ± 8.92  (9.15–36.33) [8] | 24.23 ± 6.71  (18.25–38.64) [8] |
| **Tip sclerotisation intensity (RGB scale) Mean ± SD** **(range) [n]** | 0 | 53.36 ± 9.15  (44.40–68.83) [5] | 63.36 ± 6.70  (48.80–68.58)[8] | 63.22 ± 12.79  (50.54–92.03)[8] |
| **Sensilla–upper valve** | Absent | Absent | Absent | Absent |
| **Sensilla–lower valves** | Present | Present | Present | Present |
| **Sensilla types** | 1. Campaniform type 1 (circular) | 1. Campaniform type 2  (dome elliptic)  2. Campaniform type 3  (depression)  3. Unidentified 1 (elevated structure ) | 1. Campaniform type 2  (dome elliptic)  2. Campaniform type 3  (depression)  3. Unidentified 2 (elevated structure ) | 1.Campaniform type 2  (dome elliptic)  2. Campaniform type 3  (depression)  3. Unidentified 3 (elevated structure) |

|  | ***A. agraensis*** | ***Apocrypta* sp. 2** | ***Apocrypta westwoodi*** |
| --- | --- | --- | --- |
| **Parasitism mode/Type** | Parasitoid/Inquiline/NPFW | Parasitoid/NPFW | Parasitoid/NPFW |
| **Teeth–upper valve** | Multiple teeth | Multiple teeth | Multiple teeth |
| **Teeth–lower valves** | Present | Present | Present |
| **Type of teeth** | Upper valve**–**Moderate serrations  Lower valves**–**Low serrations | Upper valve**–**Moderate serrations  Lower valves**–**Low serrations | Upper valve**–**Moderate serrations  Lower valves**–**Low serrations |
| **Tip shape–upper valve** | Notch | Notch | Notch |
| **Tip shape–lower valves** | Pointed | Pointed | Pointed |
| **Tip sclerotisation (%) Mean±SD (range) [n]** | 29.95 ± 5.78 (21.70–39.83) [8] | 42.99 ± 10.76 (24.63–61.06) [8] | 36.47 ± 7.33 (25.35–50.40) [8] |
| **Tip sclerotisation intensity (RGB scale)**  **Mean±SD** **(range) [n]** | 32.92 ± 5.56 (28.25–43.30) [8] | 24.63 ± 3.60 (20.10–30.43) [8] | 28.43 ± 3.01 (22.65–32.08) [8] |
| **Sensilla–upper valve** | Absent | Absent | Absent |
| **Sensilla–lower valves** | Present | Present | Present |
| **Sensilla types** | 1. Basiconic type 1  2. Unidentified 4 | 1. Basiconic type 2  2. Campaniform type 4  3. Campaniform type 5  4. Coeloconic type  5. Unidentified 5  6. Unidentified 6 | 1. Basiconic type 2  2. Campaniform type 4  3. Campaniform type 5  4. Coeloconic type  5. Unidentified 5  6. Unidentified 6 |

NPFW = Non-Pollinating Fig Wasp
