# Supplementary material for: Nature's Swiss Army Knives: Ovipositor Structure Mirrors Ecology in a Multitrophic Fig Wasp Community
Source: PLoS One. 2011 Aug 31;6(8):e23642. doi: 10.1371/journal.pone.0023642 (PMC3166121; doi:10.1371/journal.pone.0023642)
Supplement: Table S2 — Dimensions of sensilla observed on the lower valve of ovipositors of fig wasps of F. racemosa . (DOC) [file pone.0023642.s004.doc]

**Table S2**. Dimensions of sensilla observed on the lower valve of ovipositors of fig wasps of *F. racemosa*.

| **Type** | **Species** | **Number per valve** | **Outer diameter length (µm) (range)[n]** | **Outer diameter**  **width (µm)**  **(range)[n]** | **Inner diameter**  **length (µm) (range)[n]** | **Inner diameter**  **width (µm) (range)[n]** | **Distance from**  **tip (µm) (range)[n]** | **Description** |
| --- | --- | --- | --- | --- | --- | --- | --- | --- |
| **CS1** | *C. fusciceps* | 3  (in a triad) | 0.73±0.17  (0.43–1.00) [18] | na | 0.54 ± 0.13  (0.33–0.78) [18] | na | 6.72 ± 1.02  (5–8) [18] | Circular shape |
| **CS2** | *A. stratheni* | At least 2 | na | na | 0.56 ± 0.05  (0.53–0.60) [2] | 0.35 ± 0.03  (0.33–0.37) [2] | 4.00 ± 0.00  (4–4) [2] | Elliptic |
| **CS2** | *A. testacea* | At least 2 | na | na | 0.73 ± 0.24  (0.45–1.00) [6] | 0.27 ± 0.04  (0.22–0.33) [6] | 2.67 ± 0.52  (2–3) [6] | Elliptic |
| **CS2** | *A. fusca* | At least 2 | na | na | 0.75 ± 0.20  (0.37–1.00) [10] | 0.25 ± 0.05  (0.16–0.33) [10] | 2.40 ± 0.52  (2–3) [10] | Elliptic |
| **CS3** | *A. stratheni* | At least 4 | na | na | 0.84 ± 0.17  (0.53–1.00) [8] | 1.10 ± 1.29  (0.22–0.57) [8] | 38.88 ± 19.44  (17–74) [8] | Depression |
| **CS3** | *A. testacea* | At least 5 | na | na | 0.81 ± 0.19  (0.39–1.00) [11] | 0.20 ± 0.06  (0.13–0.33) [11] | 20.82 ± 10.30  (11–43) [11] | Depression |
| **CS3** | *A. fusca* | At least 5 | na | na | 0.91 ± 0.45  (0.40–2.00) [17] | 0.21 ± 0.08  (0.10–0.33) [17] | 25.81 ± 13.93  (11–49) [16] | Depression |
| **CS4** | *Apocrypta* sp.2 | At least 2 | 2.44 ± 0.70  (2–4) [18] | 1.05 ± 0.36  (0.58–2.00) [18] | na | na | 141.60 ± 48.72  (85–241) [10] | Shallow depression |
| **CS4** | *A. westwoodi* | At least 2 | 1.30 ± 1.33  (0.19–3.00) [7] | 0.63 ± 0.74  (0.08–2.00) [7] | na | na | 159.00 ± 21.21  (144–174) [2] | Shallow depression |
| **CS5** | *Apocrypta* sp.2 | At least 2 | 1.80 ± 0.45  (1–2) [5] | 0.93 ± 0.10  (0.80–1.00) [5] | na | na | na | Depression with shallow dome inside |
| **CS5** | *A. westwoodi* | At least 2 | 2.00 ± 0.00  (2–2) [2] | 0.98 ± 0.02  (0.97–1.00) [2] | na | na | na | Depression with shallow dome inside |
| **BS1** | *A. agraensis* | At least 4 | 0.56 ± 0.41  (0.08–1.00) [10] | 0.32 ± 0.26  (0.04–0.71) [10] | na | 0.41 ± 0.07  (0.33–0.46) [3] | 8.50 ± 7.39  (2–23) [8] | Peg |
| **Type** | **Species** | **Number** | **Outer diameter length (µm) (range)[n]** | **Outer diameter**  **width (µm) (range)[n]** | **Inner diameter**  **length (µm) (range)[n]** | **Inner diameter**  **width (µm) (range)[n]** | **Distance from**  **tip (µm) (range)[n]** | **Description** |
| **BS2** | *Apocrypta* sp.2 | At least 2 | 0.85 ± 0.13  (0.63–1.00) [11] | 0.59 ± 0.14  (0.43–0.93) [11] | 0.68 ± 0.09  (0.56–0.80) [7] | 0.32 ± 0.03  (0.25–0.35) [7] | 15.77±2.71  (12–20) [13] | Peg |
| **BS2** | *A. westwoodi* | At least 2 | 0.94 ± 0.05  (0.88–1.00) [15] | 0.57 ± 0.04  (0.49–0.64) [15] | 0.82 ± 0.12  (0.66–1.00) [9] | 0.35 ± 0.04  (0.29–0.42) [9] | 16.47±3.00  (13–22) [15] | Peg |
| **CoS** | *Apocrypta* sp.2 | At least 3 | 3.00 ± 0.67  (2.00–4.00) [28] | 1.19 ± 0.56  (0.16–2.00) [28] | na | 0.82 ± 0.32  (0.53–2.00) [20] | 121.00 ± 118.81  (54–516) [14] | Pit and peg |
| **CoS** | *A. westwoodi* | At least 3 | 2.46 ± 1.18  (0.28–4.00) [15] | 1.07 ± 0.67  (0.10–2.00) [15] | na | 0.60 ± 0.32  (0.09–0.98) [9] | 82.31 ± 21.69  (54–106) [13] | Pit and peg |
| **UN1** | *A. stratheni* | At least  30 | 2.7 ± 0.48  (2–3) [10] | 1.4 ± 0.52  (1–2) [10] | na | na | 70 [1] | Elevated structures  in a row |
| **UN2** | *A. testacea* | At least  24 | 1.1 ± 0.3  (1–2) [10] | 1.2 ± 0.48  (1–2) [10] | na | na | na | Elevated structures  in a row |
| **UN3** | *A. fusca* | At least  10 | 2.0 ± 0.6  (1–3) [7] | 0.9 ± 0.1  (0.8–1) [7] | na | na | na | Elevated structures  in a row |
| **UN4** | *A. agraensis* | At least  16 | 2.2 ± 0.4  (2–3) [10] | 1.3 ± 056  (1–2) [10] | na | na | 79 [1] | Elevated structures  in a row |
| **UN5** | *Apocrypta* sp.2 | At least 1 | 4.17 ± 0.75  (3.00–5.00) [6] | 1.26 ± 0.64  (0.88–2.00) [6] | na | 0.80 ± 0.12  (0.69–1.00) [5] | 56 ± 2.82  (54–58) [2] | Leaf-like |
| **UN5** | *A. westwoodi* | At least 1 | 2.58 ± 1.57  (0.33–4.00) [4] | 0.45 ± 0.34  (0.05–0.88) [4] | na | 0.61 ± 0.38  (0.09–0.91) [4] | 0.057 [1] | Leaf-like |
| **UN6** | *Apocrypta* sp.2 | 5.6 ± 0.55 per µm^2^ | na | na | na | na | na | Patch of pits |
| **UN6** | *A. westwoodi* | 3.4±0.55 per µm^2^ | na | na | na | na | na | Patch of pits |

CS = campaniform sensillum, BS = basiconic sensillum, CoS = coeloconic sensillum, UN = unidentified types, UN1–UN4 might be sensilla or transitional structures, na = not applicable/not available
